# RANDOMIZED CONTROLLED TRIALS FOR CANCER-RELATED COGNITIVE IMPAIRMENT IN OLDER CANCER SURVIVORS: LESSONS LEARNED

**DOI:** 10.1093/geroni/igac059.589

**Published:** 2022-12-20

**Authors:** Diane Von Ah

**Affiliations:** OSU, Columbus, Ohio, United States

## Abstract

Breast cancer survivors (BCS) face a myriad of late and long-term symptoms including cancer-related cognitive impairment (CRCI). In fact, up to 75% of the 3.8 million BCS report concerns with memory, processing information speed, and decision-making. It is hypothesized that a subset of vulnerable BCS incur ‘accelerated aging’ resulting in CRCI with older BCS at greatest risk. CRCI has many downstream negative effects on everyday functioning and health-related quality of life. Despite considerable need, there are currently no effective treatments which have been sufficiently validated for CRCI. Cognitive training, which is based on the principles of neuroplasticity (brain’s ability to reorganize and form new neural connections to accomplish tasks), may be a therapeutic option. Clinical trials from our lab and others offer insights into the needs of BCS with CRCI and considerations (facilitators, barriers, acceptability and satisfaction) for older BCS will be highlighted to address this potentially debilitating symptom.

